# (*E*)-*N*-(2,4-Dichloro­benzyl­idene)-2,5-dimeth­oxy­aniline

**DOI:** 10.1107/S1600536813005552

**Published:** 2013-03-02

**Authors:** Yong Pan, Hai-Fu Guo, De-Yun Ma

**Affiliations:** a College of Chemical Engineering, Inner Mongolia University of Technology, Inner Mongolia 010051, People’s Republic of China; b School of Chemistry and Chemical Engineering, Zhaoqing University, Zhaoqing 526061, People’s Republic of China

## Abstract

In the title compound, C_15_H_13_Cl_2_NO_2_, which was obtained by a condensation reaction of 2,5-dimeth­oxy­aniline and 2,4-dichloro­benzaldehyde, the dihedral angle between the benzene rings is 51.94 (2)°. The 2,5-dimeth­oxy­phenyl and 2,4-dichloro­phenyl groups are attached to the ends of the N=C group in an *E* conformation. Intra­molecular C—H⋯Cl and C—H⋯N contacts are observed. In the crystal, mol­ecules are linked by C—H⋯O hydrogen bonds, forming chains parallel to the *b* axis.

## Related literature

For the synthesis and applications of Schiff base–metal complexes, see: Jin *et al.* (2011 ▶). For the preparation of Schiff base compounds by the condensation reaction between 2,4-dichloro­benzaldehyde with organic amines, see: Guo *et al.* (2012 ▶). For standard bond lengths, see: Allen *et al.* (1987 ▶).
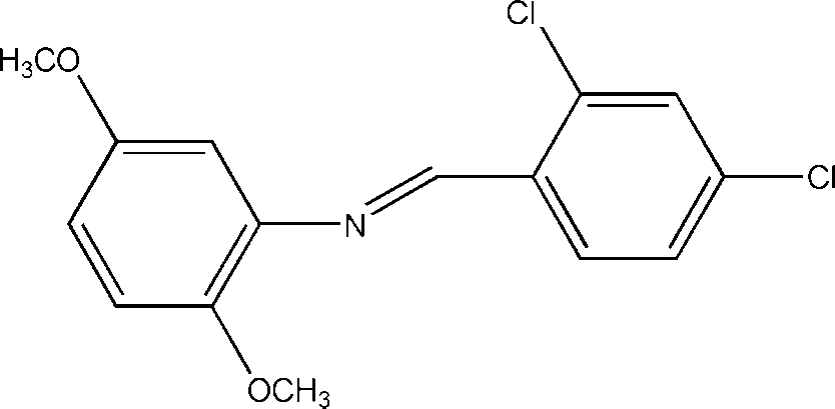


## Experimental

### 

#### Crystal data

C_15_H_13_Cl_2_NO_2_
*M* *_r_* = 310.16
Monoclinic, 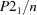

*a* = 13.2879 (12) Å
*b* = 5.1329 (5) Å
*c* = 21.1490 (18) Å
β = 96.622 (2)°
*V* = 1432.9 (2) Å^3^
*Z* = 4
Mo *K*α radiation
μ = 0.45 mm^−1^
*T* = 296 K
0.33 × 0.27 × 0.22 mm

#### Data collection

Bruker APEXII area-detector diffractometer
Absorption correction: multi-scan (*SADABS*; Sheldrick, 1996 ▶) *T*_min_ = 0.875, *T*_max_ = 0.917
7646 measured reflections
2545 independent reflections
2166 reflections with *I* > 2σ(*I*)
*R*_int_ = 0.020

#### Refinement

*R*[*F* ^2^ > 2σ(*F* ^2^)] = 0.030
*wR*(*F* ^2^) = 0.093
*S* = 1.04
2545 reflections
183 parameters
H-atom parameters constrained
Δρ_max_ = 0.18 e Å^−3^
Δρ_min_ = −0.18 e Å^−3^

### 

Data collection: *APEX2* (Bruker, 2004 ▶); cell refinement: *SAINT* (Bruker, 2004 ▶); data reduction: *SAINT*; program(s) used to solve structure: *SHELXS97* (Sheldrick, 2008 ▶); program(s) used to refine structure: *SHELXL97* (Sheldrick, 2008 ▶); molecular graphics: *XP* in *SHELXTL* (Sheldrick, 2008 ▶); software used to prepare material for publication: *SHELXL97*.

## Supplementary Material

Crystal structure: contains datablock(s) I, global. DOI: 10.1107/S1600536813005552/cq2001sup1.cif

Structure factors: contains datablock(s) I. DOI: 10.1107/S1600536813005552/cq2001Isup2.hkl

Supplementary material file. DOI: 10.1107/S1600536813005552/cq2001Isup3.cml

Additional supplementary materials:  crystallographic information; 3D view; checkCIF report

## Figures and Tables

**Table 1:** Hydrogen-bond geometry (Å, °)

*D*—H⋯*A*	*D*—H	H⋯*A*	*D*⋯*A*	*D*—H⋯*A*
C13—H13⋯O1^i^	0.93	2.62	3.357 (2)	137
C7—H7⋯Cl1	0.93	2.72	3.100 (1)	106
C13—H13⋯N1	0.93	2.52	2.826 (6)	100

Symmetry code: (i) 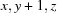.

**Fig. 1.**
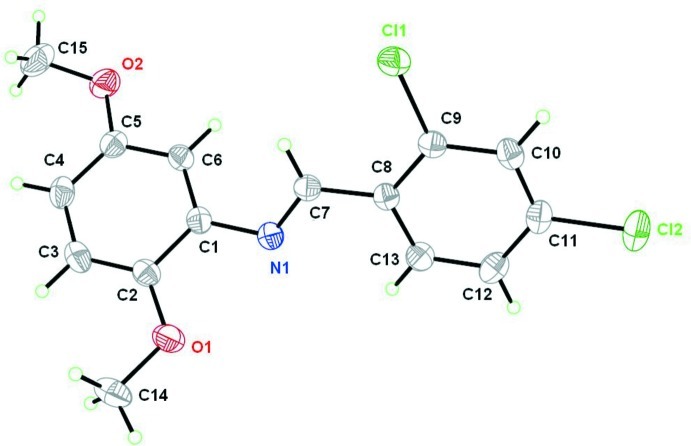
The molecular structure of the title compound, with the atomic numbering scheme. Displacement ellipsoids are drawn at the 30% probability level.

**Fig. 2.**
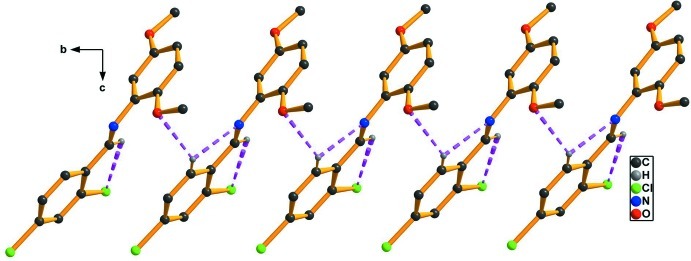
A view parallel to the a axis of crystal packing of the title compound, showing how the molecules are linked via hydrogen bonds (dashed lines). Only the H atoms involved in these interactions are shown.

## supplementary crystallographic information

### Comment

The field of Schiff bases and their complexes is rapidly developing mainly owing to facile synthesis and technological applications in many areas, such as biological activity (Jin *et al.*, 2011). As an extension of our work in the structural characterization of Schiff base compounds (Guo *et al.*, 2012), we synthesized the title compound. The title molecule, Fig. 1, has an *E* conformation around C=N double bond with a C8—C7—N1—C1 torsion angle = -174.5 (6) Å. The phenyl moiety (C1—C6/O1/O2) [maximum deviation of 0.052 (2) Å for the O2 atom] is almost planar with distances of 0.118 (3) Å (C14) and 0.298 (2) Å (C15) from the plane defined by the atoms C1—C6/O1/O2, respectively. The bond lengths (Allen *et al.*, 1987) and angles are within normal ranges and are comparable to those reported for related structures (Guo *et al.*, 2012). The dihedral angle between the substituted phenyl rings is 51.94 (2) Å. In the crystal, molecules are linked through C13—H13···O1 hydrogen bonds forming one-dimensional chains parallel to the *b* axis (Fig. 2). Moreover, intramolecular hydrogen bonding interactions are also observed (Table 1).

### Experimental

Title compound was by prepared by the condensation of 2,5-dimethoxyaniline (4.60 g, 30 mmol) with 2,4-dichlorobenzaldehyde (5.25 g, 30 mmol) in ethanol (20 ml) as the reaction medium. The solution was refluxed for 3–4 h and then allowed to cool to room temperature. The yellow precipitate was recrystallized from ethanol to give the title compound as yellow crystals. Yield 6.20 g (66.7%). [m.p. 363–365 K; ^1^H NMR(CDCl_3_, delta, p.p.m.) 8.26 (s, 1H, HC=N), 6.90–7.88 (m, 6H, Ar—H), 3.74–3.86 (m, 6H); ^13^C NMR (CDCl_3_, delta, p.p.m.) 171.5, 153.5, 144.3, 140.0, 139.7, 137.4, 131.6, 130.3, 130.5, 129.8, 127.8, 115.5, 114.1, 111.5, 58.3, 57.1, 56.7, 55.9, 55.8, 55.7].

### Refinement

All H atoms were located on the difference maps, and were treated as riding atoms with C—H distances of 0.93 and 0.96 Å, for aryl and methyl, respectively, with *U*_iso_(H) = 1.5*U*_eq_ (methyl C-atoms) and 1.2*U*_eq_(non-methyl C-atoms). The hightest peak is located 0.99 Å from Cl2 and the deepest hole is located 0.80 Å from Cl1.

### Crystal data

**Table:**

C_15_H_13_Cl_2_NO_2_	*F*(000) = 640
*M**_r_* = 310.16	*D*_x_ = 1.438 Mg m^−^^3^
Monoclinic, *P*2_1_/*n*	Mo *K*α radiation, λ = 0.71073 Å
Hall symbol: -P 2yn	Cell parameters from 5300 reflections
*a* = 13.2879 (12) Å	θ = 1.3–28.0°
*b* = 5.1329 (5) Å	µ = 0.45 mm^−^^1^
*c* = 21.1490 (18) Å	*T* = 296 K
β = 96.622 (2)°	Block, yellow
*V* = 1432.9 (2) Å^3^	0.33 × 0.27 × 0.22 mm
*Z* = 4	

### Data collection

**Table:**

Bruker APEXII area-detector diffractometer	2545 independent reflections
Radiation source: fine-focus sealed tube	2166 reflections with *I* > 2σ(*I*)
Graphite monochromator	*R*_int_ = 0.020
φ and ω scan	θ_max_ = 25.2°, θ_min_ = 1.7°
Absorption correction: multi-scan (*SADABS*; Sheldrick, 1996)	*h* = −15→15
*T*_min_ = 0.875, *T*_max_ = 0.917	*k* = −6→6
7646 measured reflections	*l* = −25→25

### Refinement

**Table:**

Refinement on *F*^2^	Primary atom site location: structure-invariant direct methods
Least-squares matrix: full	Secondary atom site location: difference Fourier map
*R*[*F*^2^ > 2σ(*F*^2^)] = 0.030	Hydrogen site location: inferred from neighbouring sites
*wR*(*F*^2^) = 0.093	H-atom parameters constrained
*S* = 1.04	*w* = 1/[σ^2^(*F*_o_^2^) + (0.0481*P*)^2^ + 0.4104*P*] where *P* = (*F*_o_^2^ + 2*F*_c_^2^)/3
2545 reflections	(Δ/σ)_max_ = 0.002
183 parameters	Δρ_max_ = 0.18 e Å^−^^3^
0 restraints	Δρ_min_ = −0.18 e Å^−^^3^

### Special details

**Table:**

Geometry. All e.s.d.'s (except the e.s.d. in the dihedral angle between two l.s. planes) are estimated using the full covariance matrix. The cell e.s.d.'s are taken into account individually in the estimation of e.s.d.'s in distances, angles and torsion angles; correlations between e.s.d.'s in cell parameters are only used when they are defined by crystal symmetry. An approximate (isotropic) treatment of cell e.s.d.'s is used for estimating e.s.d.'s involving l.s. planes.
Refinement. Refinement of *F*^2^ against ALL reflections. The weighted *R*-factor *wR* and goodness of fit *S* are based on *F*^2^, conventional *R*-factors *R* are based on *F*, with *F* set to zero for negative *F*^2^. The threshold expression of *F*^2^ > σ(*F*^2^) is used only for calculating *R*-factors(gt) *etc*. and is not relevant to the choice of reflections for refinement. *R*-factors based on *F*^2^ are statistically about twice as large as those based on *F*, and *R*- factors based on ALL data will be even larger.

### Fractional atomic coordinates and isotropic or equivalent isotropic displacement parameters (Å^2^)

**Table:**

	*x*	*y*	*z*	*U*_iso_*/*U*_eq_	
C1	0.23947 (13)	0.0926 (3)	0.90147 (8)	0.0410 (4)	
C2	0.31359 (13)	−0.0886 (4)	0.88812 (8)	0.0445 (4)	
C3	0.29198 (16)	−0.2578 (4)	0.83778 (9)	0.0534 (5)	
H3	0.3401	−0.3811	0.8295	0.064*	
C4	0.20020 (16)	−0.2487 (4)	0.79912 (9)	0.0531 (5)	
H4	0.1869	−0.3651	0.7655	0.064*	
C5	0.12894 (14)	−0.0656 (4)	0.81105 (8)	0.0479 (4)	
C6	0.14928 (14)	0.1034 (4)	0.86207 (8)	0.0449 (4)	
H6	0.1010	0.2270	0.8699	0.054*	
C7	0.19243 (13)	0.3032 (4)	0.98933 (8)	0.0413 (4)	
H7	0.1331	0.2057	0.9830	0.050*	
C8	0.20445 (12)	0.4962 (3)	1.04073 (7)	0.0384 (4)	
C9	0.13326 (13)	0.5323 (3)	1.08379 (8)	0.0395 (4)	
C10	0.14630 (14)	0.7172 (4)	1.13174 (8)	0.0448 (4)	
H10	0.0982	0.7379	1.1600	0.054*	
C11	0.23178 (15)	0.8692 (4)	1.13661 (8)	0.0460 (4)	
C12	0.30436 (16)	0.8415 (4)	1.09520 (9)	0.0526 (5)	
H12	0.3621	0.9453	1.0993	0.063*	
C13	0.28939 (14)	0.6569 (4)	1.04783 (8)	0.0480 (4)	
H13	0.3377	0.6390	1.0196	0.058*	
C14	0.47361 (16)	−0.2867 (5)	0.92139 (12)	0.0679 (6)	
H14A	0.4417	−0.4528	0.9253	0.102*	
H14B	0.5300	−0.2699	0.9538	0.102*	
H14C	0.4971	−0.2736	0.8802	0.102*	
C15	0.0017 (2)	−0.2339 (6)	0.73297 (11)	0.0803 (8)	
H15A	0.0448	−0.2478	0.6996	0.121*	
H15B	−0.0663	−0.1967	0.7148	0.121*	
H15C	0.0028	−0.3952	0.7560	0.121*	
Cl1	0.02571 (3)	0.33712 (10)	1.08021 (2)	0.05366 (16)	
Cl2	0.24982 (5)	1.10453 (10)	1.19606 (2)	0.06520 (19)	
N1	0.26115 (11)	0.2661 (3)	0.95328 (7)	0.0441 (4)	
O1	0.40247 (10)	−0.0847 (3)	0.92844 (7)	0.0575 (4)	
O2	0.03701 (11)	−0.0305 (3)	0.77496 (7)	0.0662 (4)	

### Atomic displacement parameters (Å^2^)

**Table:**

	*U*^11^	*U*^22^	*U*^33^	*U*^12^	*U*^13^	*U*^23^
C1	0.0453 (9)	0.0381 (10)	0.0413 (9)	−0.0026 (7)	0.0125 (7)	0.0004 (7)
C2	0.0446 (10)	0.0457 (11)	0.0454 (9)	0.0017 (8)	0.0139 (7)	0.0044 (8)
C3	0.0615 (12)	0.0460 (11)	0.0561 (11)	0.0066 (9)	0.0211 (9)	−0.0026 (9)
C4	0.0670 (13)	0.0485 (12)	0.0461 (10)	−0.0092 (9)	0.0164 (9)	−0.0087 (9)
C5	0.0491 (10)	0.0535 (12)	0.0421 (9)	−0.0095 (9)	0.0100 (8)	−0.0005 (8)
C6	0.0436 (10)	0.0477 (11)	0.0449 (9)	0.0010 (8)	0.0113 (7)	−0.0028 (8)
C7	0.0404 (9)	0.0417 (10)	0.0413 (8)	0.0014 (7)	0.0034 (7)	0.0020 (8)
C8	0.0423 (9)	0.0361 (9)	0.0365 (8)	0.0048 (7)	0.0024 (7)	0.0037 (7)
C9	0.0400 (9)	0.0377 (9)	0.0403 (8)	0.0047 (7)	0.0016 (7)	0.0062 (7)
C10	0.0535 (11)	0.0430 (10)	0.0376 (9)	0.0129 (8)	0.0046 (7)	0.0027 (8)
C11	0.0643 (12)	0.0354 (10)	0.0357 (8)	0.0070 (8)	−0.0054 (8)	0.0023 (7)
C12	0.0587 (11)	0.0481 (11)	0.0491 (10)	−0.0106 (9)	−0.0019 (8)	0.0025 (9)
C13	0.0496 (10)	0.0517 (12)	0.0435 (9)	−0.0029 (9)	0.0090 (8)	0.0014 (8)
C14	0.0483 (12)	0.0714 (15)	0.0875 (16)	0.0155 (10)	0.0219 (11)	0.0111 (13)
C15	0.0758 (16)	0.102 (2)	0.0615 (13)	−0.0301 (15)	0.0016 (11)	−0.0213 (14)
Cl1	0.0457 (3)	0.0577 (3)	0.0593 (3)	−0.0027 (2)	0.0129 (2)	−0.0005 (2)
Cl2	0.0955 (4)	0.0480 (3)	0.0478 (3)	0.0076 (3)	−0.0101 (2)	−0.0082 (2)
N1	0.0450 (8)	0.0451 (9)	0.0425 (8)	0.0008 (7)	0.0065 (6)	−0.0023 (7)
O1	0.0463 (7)	0.0635 (9)	0.0628 (8)	0.0102 (6)	0.0072 (6)	−0.0008 (7)
O2	0.0571 (9)	0.0824 (11)	0.0569 (8)	−0.0062 (8)	−0.0026 (6)	−0.0149 (8)

### Geometric parameters (Å, º)

**Table:**

C1—C6	1.380 (2)	C9—C10	1.385 (3)
C1—C2	1.407 (2)	C9—Cl1	1.7397 (18)
C1—N1	1.416 (2)	C10—C11	1.372 (3)
C2—O1	1.375 (2)	C10—H10	0.9300
C2—C3	1.379 (3)	C11—C12	1.383 (3)
C3—C4	1.389 (3)	C11—Cl2	1.7399 (18)
C3—H3	0.9300	C12—C13	1.376 (3)
C4—C5	1.378 (3)	C12—H12	0.9300
C4—H4	0.9300	C13—H13	0.9300
C5—O2	1.376 (2)	C14—O1	1.423 (2)
C5—C6	1.387 (3)	C14—H14A	0.9600
C6—H6	0.9300	C14—H14B	0.9600
C7—N1	1.270 (2)	C14—H14C	0.9600
C7—C8	1.466 (2)	C15—O2	1.415 (3)
C7—H7	0.9300	C15—H15A	0.9600
C8—C13	1.392 (3)	C15—H15B	0.9600
C8—C9	1.399 (2)	C15—H15C	0.9600
			
C6—C1—C2	119.02 (16)	C11—C10—C9	118.51 (16)
C6—C1—N1	121.81 (16)	C11—C10—H10	120.7
C2—C1—N1	119.12 (16)	C9—C10—H10	120.7
O1—C2—C3	125.15 (17)	C10—C11—C12	121.70 (17)
O1—C2—C1	115.92 (16)	C10—C11—Cl2	119.53 (14)
C3—C2—C1	118.91 (17)	C12—C11—Cl2	118.77 (15)
C2—C3—C4	121.60 (18)	C13—C12—C11	118.63 (18)
C2—C3—H3	119.2	C13—C12—H12	120.7
C4—C3—H3	119.2	C11—C12—H12	120.7
C5—C4—C3	119.38 (18)	C12—C13—C8	122.25 (17)
C5—C4—H4	120.3	C12—C13—H13	118.9
C3—C4—H4	120.3	C8—C13—H13	118.9
O2—C5—C4	124.94 (17)	O1—C14—H14A	109.5
O2—C5—C6	115.51 (17)	O1—C14—H14B	109.5
C4—C5—C6	119.53 (18)	H14A—C14—H14B	109.5
C1—C6—C5	121.49 (17)	O1—C14—H14C	109.5
C1—C6—H6	119.3	H14A—C14—H14C	109.5
C5—C6—H6	119.3	H14B—C14—H14C	109.5
N1—C7—C8	121.49 (17)	O2—C15—H15A	109.5
N1—C7—H7	119.3	O2—C15—H15B	109.5
C8—C7—H7	119.3	H15A—C15—H15B	109.5
C13—C8—C9	116.89 (16)	O2—C15—H15C	109.5
C13—C8—C7	119.87 (15)	H15A—C15—H15C	109.5
C9—C8—C7	123.23 (16)	H15B—C15—H15C	109.5
C10—C9—C8	122.02 (17)	C7—N1—C1	117.58 (15)
C10—C9—Cl1	117.34 (13)	C2—O1—C14	117.26 (17)
C8—C9—Cl1	120.61 (14)	C5—O2—C15	117.40 (19)

### Hydrogen-bond geometry (Å, º)

**Table:**

*D*—H···*A*	*D*—H	H···*A*	*D*···*A*	*D*—H···*A*
C13—H13···O1^i^	0.93	2.62	3.357 (2)	137
C7—H7···Cl1	0.93	2.72	3.100 (1)	106
C13—H13···N1	0.93	2.52	2.826 (6)	100

Symmetry code: (i) *x*, *y*+1, *z*.
